# Efficacy and safety of monotherapy with the novel sodium/glucose cotransporter-2 inhibitor tofogliflozin in Japanese patients with type 2 diabetes mellitus: a combined Phase 2 and 3 randomized, placebo-controlled, double-blind, parallel-group comparative study

**DOI:** 10.1186/1475-2840-13-65

**Published:** 2014-03-28

**Authors:** Kohei Kaku, Hirotaka Watada, Yasuhiko Iwamoto, Kazunori Utsunomiya, Yasuo Terauchi, Kazuyuki Tobe, Yukio Tanizawa, Eiichi Araki, Masamichi Ueda, Hideki Suganami, Daisuke Watanabe

**Affiliations:** 1 Department of Internal Medicine, Kawasaki Medical School, 577 Matsushima, Kurashiki, Okayama 701-0192, Japan; 2 Department of Endocrinology & Metabolism, Juntendo University Graduate School of Medicine, 2-1-1, Hongo, Bunkyo-ku, Tokyo 113-8421, Japan; 3 Tokyo Women's Medical University School of Medicine, 8-1, Kawada, Shinjuku-ku, Tokyo 162-8666, Japan; 4 Division of Diabetes, Metabolism and Endocrinology, Department of Internal Medicine, Jikei University School of Medicine, 3-25-8, Nishi-Shimbashi, Minato-ku, Tokyo 105-8461, Japan; 5 Department of Endocrinology and Metabolism, Graduate School of Medicine, Yokohama City University, 3-9 Fukuura, Kanazawa-ku, Yokohama 236-0004, Japan; 6 The First Department of Internal Medicine, University of Toyama, 2630 Sugitani, Toyama 930-0194, Japan; 7 Division of Endocrinology, Metabolism, Hematological Science and Therapeutics, Yamaguchi University Graduate School of Medicine, 1-1-1 Minami-Kogushi, Ube, Yamaguchi, 755-8505, Japan; 8 Department of Metabolic Medicine, Faculty of Life Sciences, Kumamoto University, 2-39-1 Kurokami, Kumamoto 860-8555, Japan; 9 Clinical Research Planning Department, Chugai, 1-1 Nihonbashi-Muromachi 2-Chome, Chuo-ku, Tokyo 103-8324, Japan; 10 Clinical Data Science Department, Biostatistics Section, Kowa, 3-4-14, Nihonbashi-honcho, Chuo-ku, Tokyo 103-0023, Japan; 11 Research & Development, Clinical Sciences & Operations, Biostatistics & Programming, Biostatistics, Sanofi, Tokyo Opera City Tower, 3-20-2, Nishi Shinjuku, Shinjuku-ku, Tokyo 163-1488, Japan

**Keywords:** SGLT2 inhibitor, Tofogliflozin, CSG452, RG7201, HbA_1c_, Body weight

## Abstract

**Background:**

In recent years, several oral antidiabetic drugs with new mechanisms of action have become available, expanding the number of treatment options. Sodium/glucose cotransporter-2 (SGLT2) inhibitors are a new class of oral antidiabetic drugs with an insulin-independent mechanism promoting urinary glucose excretion. We report the results of a combined Phase 2 and 3 clinical study (Japic CTI-101349) of the SGLT2 inhibitor tofogliflozin (CSG452, RG7201) in Japanese patients with type 2 diabetes mellitus.

**Methods:**

The efficacy and safety of tofogliflozin were assessed in this multicenter, placebo-controlled, randomized, double-blind parallel-group study involving 230 patients with type 2 diabetes mellitus with inadequate glycemic control on diet/exercise therapy. Between 30 October 2010 and 28 February 2012, patients at 33 centers were randomized to either placebo (n = 56) or tofogliflozin (10, 20, or 40 mg; n = 58 each) orally, once daily for 24 weeks. The primary efficacy endpoint was the change from baseline in HbA_1c_ at week 24.

**Results:**

Overall, 229 patients were included in the full analysis set (placebo: n = 56; tofogliflozin 10 mg: n = 57; tofogliflozin 20 and 40 mg: n = 58 each). The least squares (LS) mean change (95% confidence interval) from baseline in HbA_1c_ at week 24 was −0.028% (−0.192 to 0.137) in the placebo group, compared with −0.797% (−0.960 to −0.634) in the tofogliflozin 10 mg group, −1.017% (−1.178 to −0.856) in the tofogliflozin 20 mg group, and −0.870% (−1.031 to −0.709) in the tofogliflozin 40 mg group (p < 0.0001 for the LS mean differences in all tofogliflozin groups vs placebo). There were also prominent decreases in fasting blood glucose, 2-h postprandial glucose, and body weight in all tofogliflozin groups compared with the placebo group. The main adverse events were hyperketonemia, ketonuria, and pollakiuria. The incidence of hypoglycemia was low. Furthermore, most adverse events were classified as mild or moderate in severity.

**Conclusions:**

Tofogliflozin 10, 20, or 40 mg administered once daily as monotherapy significantly decreased HbA_1c_ and body weight, and was generally well tolerated in Japanese patients with type 2 diabetes mellitus. Phase 3 studies were recently completed and support the findings of this combined Phase 2 and 3 study.

**Trial registration:**

This study was registered in the JAPIC clinical trials registry (ID: Japic CTI-101349).

## Introduction

In recent years, antidiabetic drugs with new mechanisms of action have become available, expanding the treatment options [1]. Some antidiabetic drugs, such as insulin, sulfonylureas, and glinides, are associated with relatively high rates of hypoglycemia that may limit their use in some patients [2]. In addition, insulin secretagogues may cause weight gain, which can exacerbate insulin resistance. Even in patients who achieve good glycemic control with antidiabetic drugs as monotherapy or combination therapy, the pathological progression of diabetes may lead to a gradual worsening of glycemic control, and further increases in the doses of the antidiabetic drug(s) may not allow the patient to regain glycemic control [1]. Finally, many classes of antihyperglycemic agents are directly or indirectly reliant on insulin, with the main exceptions being metformin, amylin mimetics, and α-glucosidase inhibitors [2]. Therefore, there is a need for new insulin-independent antidiabetic drugs that have a low risk of hypoglycemia, do not increase body weight, and improve the clinical features of diabetes mellitus.

Sodium/glucose cotransporter-2 (SGLT2) inhibitors represent a novel class of antidiabetic drugs that might satisfy these requirements. Inhibition of SGLT2 represents a new mechanism of action because it reduces blood glucose levels by inhibiting renal glucose reabsorption and increasing urinary excretion of excess glucose [3]. Several members of this class of drugs were recently approved for the treatment of type 2 diabetes, including canagliflozin in the US, dapagliflozin in Europe, and ipragliflozin in Japan. SGLT2 inhibitors could also be combined with currently available oral antidiabetic drugs that have different mechanisms of action [3]. Tofogliflozin (CSG452, RG7201) is a highly selective inhibitor SGLT2 that is under development for the treatment of type 2 diabetes mellitus. As part of its development, the Tofogliflozin 003 combined Phase 2 and 3 Study (CSG003JP) was designed to assess the efficacy and safety of tofogliflozin in patients with type 2 diabetes mellitus with inadequate glycemic control on diet/exercise therapy using a placebo-controlled, randomized, double-blind, parallel-group design. Efficacy was assessed based on the change in HbA_1c_ from baseline to week 24. We also assessed the dose-dependent effects of tofogliflozin on the change in HbA_1c_ and other secondary endpoints. The study was conducted in accordance with the “Guidelines for Clinical Evaluation of Oral Hypoglycemic Agents” in Japan for Phase 2 and 3 studies [4].

## Methods

### Study design

**Figure 1:**
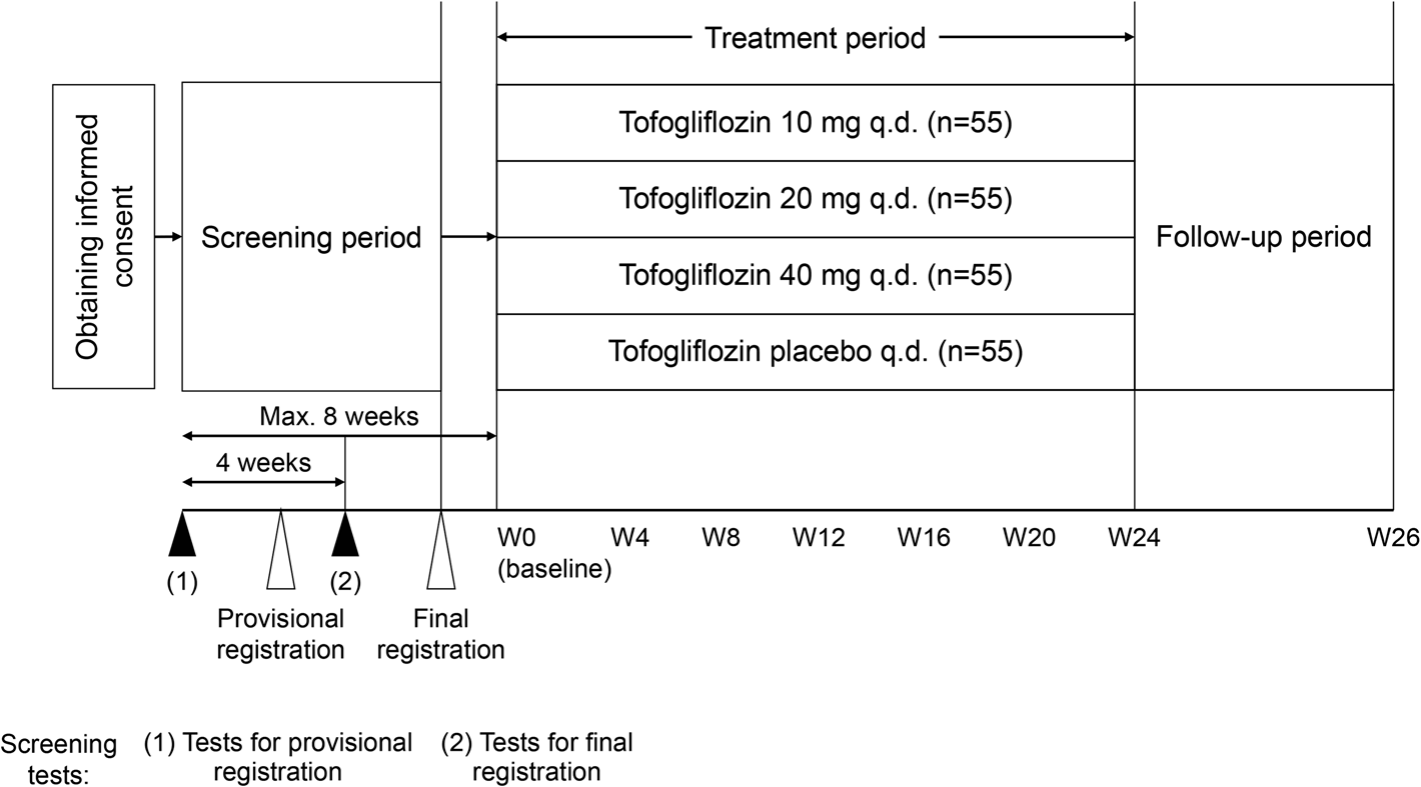
**Study design.** Patients underwent initial screening tests and those who met the inclusion criteria were provisionally registered. Patients then entered a second screening phase and those who still met the inclusion criteria were accepted for final registration and were randomized. The overall screening period was 8 weeks. An additional visit was arranged for patients who discontinued the study.

This was a multicenter, randomized, placebo-controlled, double-blind, parallel-group study performed at 33 specialist and non-specialist hospitals and clinics spread throughout Japan. The study was conducted in accordance with the Declaration of Helsinki, Good Clinical Practice, the International Conference on Harmonization of Technical Requirements for Registration of Pharmaceuticals for Human Use guidelines, and Japanese law. The study was approved by ethics committees/institutional review boards at all of the participating institutions. All patients provided informed consent prior to being enrolled. After providing informed consent, patients underwent initial screening and those who met the inclusion criteria were provisionally registered. Patients then entered a second screening phase and those who still met the inclusion criteria were accepted for final registration and were randomized at a 1:1:1:1 ratio to the four study groups. An investigator at the registration center created and managed a random allocation table, and confirmed that the study drugs and packaging were indistinguishable. After confirmation of eligibility, the patients were allocated to the study groups by the registration center using a dynamic, minimization method, with stratification for HbA_1c_ (JDS units: <8.0% or ≥8.0%) and sex. The participating clinicians at each center gave the allocated drugs to the individual patients. Patient allocation was managed using a central web-based system. To further maintain blinding, investigators were not permitted to measure urinary glucose levels or drug concentrations. The investigator responsible for managing the allocation table could only reveal the allocations after the data had been reported. The overall screening period was 8 weeks. Patients were then randomized to receive tofogliflozin 10, 20, or 40 mg, or placebo, which were to be administered orally, once daily before breakfast for 24 weeks after the screening period (Figure 1). All patients took two tablets to make up the required dose: (1) one tofogliflozin or placebo 10 or 20 mg tablet; and (2) one tofogliflozin or placebo 40 mg tablet. The tofogliflozin and placebo 10 and 20 mg tablets were identical in appearance, as were the tofogliflozin and placebo 40 mg tablets. For example, patients in the 10 mg group took one tofogliflozin 10 mg tablet plus one placebo 40 mg tablet. Visits were scheduled at weeks 0, 4, 8, 12, 16, 20, and 24 during treatment. A visit was also arranged at the time of discontinuation from the study. The tests and examinations performed at each visit are listed in Additional file 1: Table S1.

### Inclusion and exclusion criteria

Patients were aged 20–74 years and had been diagnosed with type 2 diabetes mellitus. Patients were included if they met the following criteria: (1) treatment with diet and exercise therapy only for ≥8 weeks before screening for provisional registration; (2) HbA_1c_ at screening of ≥7.3 to <10.3% (National Glycohemoglobin Standardization Program [NGSP] values) and body mass index (BMI) at screening of ≥18.5 to <45.0 kg/m^2^; (3) percent changes in HbA_1c_ (% change in Japan Diabetes Society [JDS] units, values presented in text are NGSP units) and body weight from the provisional registration visit to the final registration visit of ≤10% and <5%, respectively; (4) controlled blood pressure and, in patients who were concomitantly using antihypertensive drugs, only patients who did not need to change their dosing regimens. Patients using other antidiabetic drugs were eligible providing their prior drug was stopped ≥8 weeks before the provisional registration. HbA_1c_ was originally reported in JDS units and was converted to NGSP units using the following equation: HbA_1c_ (NGSP) = HbA_1c_ (JDS value)/0.999 + 0.288.

The main exclusion criteria were: (1) type 1 diabetes mellitus; (2) acute diabetic complications (e.g., diabetic ketoacidosis), acute coronary syndromes, stroke, or genitourinary infections within 24 weeks before the screening for provisional registration; (3) dehydration requiring hospitalization within 12 weeks before the screening for provisional registration; and (4) severe or frequent episodes of hypoglycemia within 4 weeks before the screening for provisional registration. Patients with uncontrolled blood pressure, acute coronary syndrome, or stroke, for example, were excluded to avoid potential worsening of these conditions during treatment with an experimental drug. Patients with a history of infection or hypoglycemia were excluded because of the difficulty in evaluating the safety of tofogliflozin.

### Efficacy

The primary efficacy endpoint was the change from baseline in HbA_1c_ at week 24. If a patient did not have a recorded HbA_1c_ value at week 24, the last post-baseline value measured before the scheduled visit at week 24 was used as the value at week 24 (last observation carried forward [LOCF] procedure). The secondary efficacy endpoints included the changes from baseline to week 24 for fasting plasma glucose; 2-h postprandial glucose (after a meal tolerance test); fasting and 2-h postprandial insulin (after a meal tolerance test); glycoalbumin; body weight; pancreatic β cell function (homeostatic model assessment [HOMA-β]) and insulin resistance (HOMA-IR) and Matsuda Index; insulin, serum lipid levels (total cholesterol, low-density lipoprotein–cholesterol (LDL-C), high-density lipoprotein–cholesterol, and triglycerides); adiponectin; blood pressure; and waist circumference. All secondary efficacy endpoints were assessed using the LOCF procedure.

### Safety

Safety endpoints included adverse events (AEs), which included subjective symptoms and objective findings, laboratory tests, physical findings, vital signs, 12-lead electrocardiography, and self-monitored blood glucose. Adverse events (AEs) were coded using the MedDRA (Ver. 13.1). For AEs that occurred after administration of study drug, the following information was recorded by an investigator in a case report form: type of AE, degree of severity, date of occurrence, date of restoration/remission, treatment, outcome, and relationship to the study drug (or evidence for the lack of relationship if the study drug was not related to the event). If an investigator observed serious AEs occurring during the study or within 2 weeks after terminating the study drug administration, whether related to the study drug or not, they reported the events to the sponsor or emergency center by phone or fax as soon as possible (within 24 hours of observing the serious AE). Adverse events for which a causal relationship could not be ruled out were defined as adverse drug reactions (ADRs). All instances of hypoglycemia were reported as AEs. Hypoglycemia was defined as either (1) signs/symptoms consistent with hypoglycemia that resolved after ingesting food or administration of glucagon or glucose, or (2) blood glucose ≤ 50 mg/dL with or without signs/symptoms of hypoglycemia.

Blood biochemical (including efficacy variables) and laboratory variables were measured using standardized, validated methods at an accredited central laboratory (Mitsubishi Chemical Medience Corp., Tokyo, Japan). The blood tofogliflozin concentration was measured by Swiss BioAnalytics AG (Birsfelden, Switzerland).

### Statistical analysis

For each treatment group, patient demographics are summarized with appropriate descriptive statistics (means and standard deviation [SD] for continuous variables, and counts and percentages for categorical variables).

The sample size was calculated based on the expected changes in HbA_1c_ in the placebo and 20 mg tofogliflozin groups of −0.2% and −0.7%, respectively. With a common SD of 0.75% based on the results of a prior Phase 2 study (unpublished data), 49 patients were needed per group to show superiority of tofogliflozin over placebo at a power of 90% and two-sided *P* < 0.05. With an expected dropout rate of 10%, the target sample size was 55 patients in each group.

The primary endpoint (change from baseline in HbA_1c_ at week 24) was analyzed using an analysis of covariance (ANCOVA) model with treatment groups (placebo and tofogliflozin 10, 20, or 40 mg) as a fixed effect and baseline HbA_1c_ and gender as covariates to verify the superiority of tofogliflozin over placebo. To control the overall significance level, a closed testing procedure using the assumption of a monotonic dose-response curve was applied. Once the superiority of tofogliflozin 40 mg over placebo was verified, tests for the superiority of tofogliflozin 20 and 10 mg were performed. The (two-sided) significance level for each test was 0.05. The secondary endpoints body weight and fasting plasma glucose (FPG) were analyzed using an ANCOVA model with treatment groups (placebo and tofogliflozin 10, 20, or 40 mg) as a fixed effect and the corresponding baseline value as a covariate. Other secondary endpoints are presented descriptively as the mean ± SD or n (%), as appropriate. Continuous variables were compared using *t* tests. In this article, all HbA_1c_ results are presented using NGSP values.

The number of patients with AEs and the number of AEs/ADRs were tabulated by system organ class and preferred term. Changes over time in laboratory values are presented descriptively and laboratory variables were studied for any abnormal changes of ≥5%.

Efficacy analyses were conducted in the full analysis set, which was defined as all randomized patients who received at least one dose of the study drug and had both a baseline value and at least one post baseline value of HbA_1c_. The efficacy analyses were repeated in the per-protocol set, which was defined as all randomized patients who completed the trial without a major protocol violation. Safety analyses were conducted in the safety analysis set, which was defined as all randomized patients who received at least one dose of the study drug.

## Results

### Patient disposition

A total of 235 Japanese patients were randomized (57 in the placebo group, 59 in the tofogliflozin 10 mg group, 60 in the tofogliflozin 20 mg group, and 59 in the tofogliflozin 40 mg group). The study was performed between 30 October 2010 (first patient enrolled) and 28 February 2012 (final visit of the last patient). Table 1 shows the number of patients included in the safety analysis set, full analysis set (FAS), and per-protocol set (PPS).

**Table 1:** Numbers of patients included in each analysis set, reasons for study withdrawal, and numbers of patients who completed the study

**Analysis set**	**Placebo**	**Tofogliflozin**			
		**10 mg**	**20 mg**	**40 mg**	**Total**
Target sample size	55	55	55	55	220
Patients enrolled	57	59	60	59	235
SAS*	56	58	58	58	230
Patients excluded from the SAS (did not take the study drug)	1	1	2	1	5
FAS^†^	56	57	58	58	229
Patients excluded from the FAS	0	1^§^	0	0	1
PPS^‡^	54	54	58	56	222
Patients excluded from the PPS	2	3	0	2	7
Administered <75% of the scheduled doses	0	0	0	1	1
Administered the study drug for <8 weeks from the start of treatment	2	2	0	1^§‖^	5
Used a prohibited drug during treatment	0	1	0	1^‖^	2
Study withdrawals before starting the study drug	1	1	2	1	5
Violated eligibility criteria	0	0	1	0	1
Other protocol violation	0	0	0	1	1
Patient decided to withdraw	0	1	1	0	2
Administrative/other reason	1	0	0	0	1
Study withdrawals after starting the study drug	8	4	1	4	17
Adverse event	1	2	0	3	6
Inadequate efficacy	4	0	0	0	4
Protocol violation	0	1	0	1	2
Patient did not administer the study drug/did not cooperate	2	1	0	0	3
Patient decided to withdraw	0	0	1	0	1
Other reason	1	0	0	0	1
Completers	48	54	57	54	213

*The safety analysis set was defined as all randomized patients who received at least one dose of the study drug.

^†^The full analysis set was defined as all randomized patients who received at least one dose of the study drug and had both a baseline value and at least one post baseline value of HbA_1c_.

^‡^The per-protocol set defined as all randomized patients who completed the trial without a major protocol violation.

^§^No post-baseline HbA_1c_ measurement.

^‖^This was the same patient.

Abbreviations: *SAS* safety analysis set, *FAS* full analysis set, *PPS* per-protocol set.

Table 2 shows the patient characteristics for the FAS. The range of means (for continuous variables) or proportions (for categorical variables) for key characteristics across treatment groups was as follows. Female patients accounted for 32.8–33.9% of all patients, the mean age was 56.6–58.6 years, and 22.4–31.6% of patients were aged ≥65 years. The mean body weight was 67.3–71.2 kg, and the mean BMI was 25.0–26.0 kg/m^2^. The mean duration of type 2 diabetes mellitus was 6.0–6.7 years, and 26.3–39.7% of patients had previously received treatments for diabetes mellitus. The mean HbA_1c_ was 8.34–8.45%, and the mean fasting blood glucose was 167.9–170.2 mg/dL. The patient characteristics were generally comparable among the treatment groups.

**Table 2:** Patient characteristics at baseline (FAS)

		**Placebo**	**Tofogliflozin**		
			**10 mg**	**20 mg**	**40 mg**
n		56	57	58	58
Age (years), Mean (SD)		56.8 (9.9)	58.6 (9.8)	56.6 (10.2)	57.0 (9.1)
Gender, n (%)	Male	37 (66.1%)	38 (66.7%)	39 (67.2%)	39 (67.2%)
	Female	19 (33.9%)	19 (33.3%)	19 (32.8%)	19 (32.8%)
Body weight (kg), Mean (SD)		71.20 (12.64)	67.26 (12.67)	68.06 (15.82)	68.72 (11.91)
BMI (kg/m^2^), Mean (SD)		26.00 (4.11)	25.07 (3.53)	24.99 (4.55)	25.78 (4.10)
HbA_1c_ (%), Mean (SD)		8.41 (0.78)	8.45 (0.75)	8.34 (0.81)	8.37 (0.77)
Fasting plasma glucose (mg/dL), Mean (SD)		168.8 (24.9)	170.2 (32.4)	168.7 (29.6)	167.9 (37.0)
eGFR (mL/min/1.73 m^2^), Mean (SD)		83.78 (17.68)	84.90 (20.15)	86.78 (19.62)	86.00 (18.18)
Systolic blood pressure (mmHg), Mean (SD)		128.3 (13.7)	128.0 (15.6)	131.3 (12.9)	129.3 (12.5)
Diastolic blood pressure (mmHg), Mean (SD)		76.7 (11.4)	78.6 (10.6)	79.2 (11.7)	78.7 (9.0)
Duration of diabetes (years), Mean (SD)		6.0 (6.1)	6.3 (7.1)	6.4 (5.1)	6.7 (5.5)
Prior treatment of diabetes	Yes	16 (28.6%)	15 (26.3%)	23 (39.7%)	16 (27.6%)
	No	40 (71.4%)	42 (73.7%)	35 (60.3%)	42 (72.4%)

Abbreviations: *BMI* body mass index, *eGFR* estimated glomerular filtration rate.

### Change in HbA_1c_ from baseline to week 24

**Figure 2:**
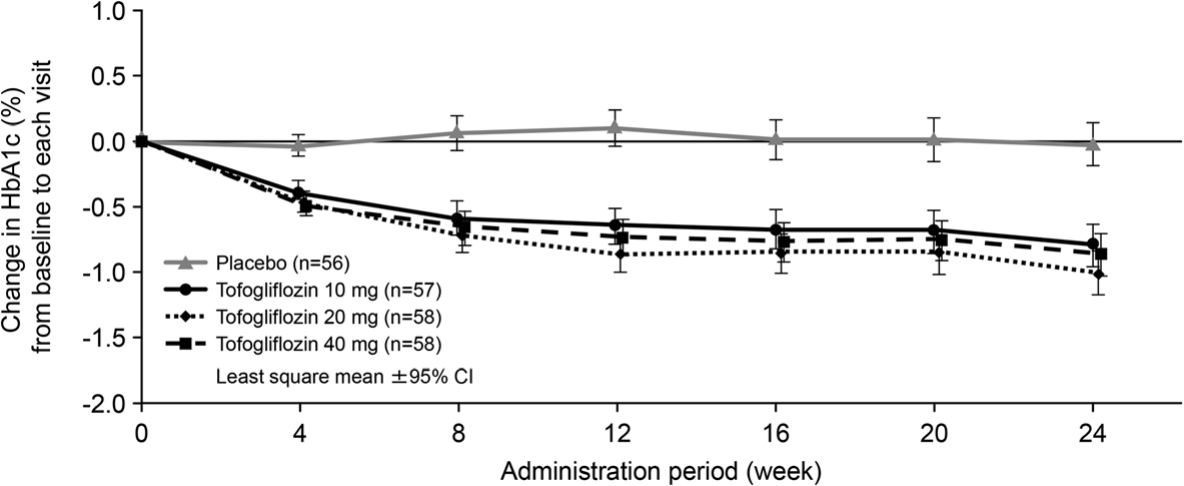
**Mean change (LS mean) in HbA**_**1c **_**from baseline to time of visit.** Mean changes (LS mean and 95% CI) in HbA_1c_ from baseline to time of visit for each treatment group (placebo, tofogliflozin 10, 20 or 40 mg) in the FAS (with LOCF) were plotted. Mean change from baseline in HbA_1c_ at week 24 (LS mean) and LS mean difference versus placebo are shown in the table below the graph. LS means, LS mean differences and 95% CI were estimated using an ANCOVA model with treatment groups (placebo and tofogliflozin 10, 20, or 40 mg) as a fixed effect and baseline HbA_1c_ and gender as covariates.

Table 3 presents the changes from baseline in HbA_1c_ at week 24. The least squares (LS) mean change (95% confidence interval [CI]) in HbA_1c_ from baseline to week 24 was significantly greater in all three tofogliflozin groups than in the placebo group, with placebo-adjusted mean changes of −0.769%, −0.990%, and −0.842% in the tofogliflozin 10 mg, 20 mg, and 40 mg groups, respectively. The decrease in HbA_1c_ from baseline to week 24 was significantly greater (*P* < 0.0001) in all three tofogliflozin groups compared with the placebo group, with the tofogliflozin 20 mg group showing the greatest decrease throughout the study (Figure 2).

**Table 3:** **Changes in primary and secondary endpoints from baseline to week 24 and proportion of patients with target HbA**_**1c **_**levels at week 24 (FAS)**

**Variable**	**Placebo**	**Tofogliflozin**		
		**10 mg**	**20 mg**	**40 mg**
HbA_1c_ (%)				
N	56	57	58	58
LS mean (95% CI)	−0.028 (−0.192 to 0.137)	−0.797 (−0.960 to −0.634)^†††^	−1.017 (−1.178 to −0.856)^†††^	−0.870 (−1.031 to −0.709)^†††^
Placebo-adjusted difference	—	−0.769	−0.990	−0.842
Fasting blood glucose (mg/dL)				
N	56	57	58	58
LS mean (95% CI)	−8.561 (−13.247 to −3.875)	−31.868 (−36.514 to −27.222)^†††^	−35.899 (−40.504 to −31.294)^†††^	−32.327 (−36.933 to −27.722)^†††^
Placebo-adjusted difference	—	−23.307	−27.338	−23.766
Body weight (kg)				
N	56	57	58	58
LS mean (95% CI)	−0.356 (−0.836 to 0.123)	−2.230 (−2.704 to −1.756)^†††^	−2.851 (−3.320 to −2.382)^†††^	−2.971 (−3.440 to −2.502)^†††^
Placebo-adjusted difference	—	−1.87	−2.50	−2.61
Glycoalbumin (%)				
N	53	55	58	58
Mean (SD)	0.19 (2.07)	−3.09 (2.19)***^†††^	−3.52 (2.84)***^†††^	−3.01 (2.33)***^†††^
2-h PPG (mg/dL)				
N	48	53	56	53
Mean (SD)	−3.33 (47.6)	−63.5 (49.0)***^†††^	−71.0 (63.7)***^†††^	−60.2 (47.2)***^†††^
Fasting insulin (μU/mL)				
N	48	52	56	57
Mean (SD)	−0.44 (4.59)	−1.36 (2.95)**	−2.12 (5.81)**	−2.01 (2.80)***^†^
2-h postprandial insulin (μU/mL)				
N	45	50	56	54
Mean (SD)	−0.26 (8.81)	−4.14 (10.59)**	−7.25 (13.74)***^††^	−5.58 (7.73)***^††^
HOMA-β				
N	48	52	56	57
Mean (SD)	−0.26 (15.62)	5.15 (13.48)**	3.97 (26.70)	2.67 (12.90)
HOMA − IR				
N	48	52	56	57
Mean (SD)	−0.296 (2.212)	−1.136 (1.532)***^†^	−1.469 (2.407)***^†^	−1.345 (1.601)*** ^††^
Matsuda Index				
N	40	45	50	49
Mean (SD)	0.434 (2.764)	2.137 (2.447)***^††^	2.233 (2.339)***^††^	2.351 (1.954)***^†††^
**Serum lipids**				
Total cholesterol (mg/dL)				
N	53	55	58	58
Mean (SD)	−6.8 (26.6)	−2.5 (20.6)	−3.1 (28.4)	1.1 (23.8)
LDL-cholesterol (mg/dL)				
N	53	55	58	58
Mean (SD)	−4.3 (21.6)	−2.7 (19.8)	−2.2 (24.0)	4.5 (21.9)^†^
HDL-cholesterol (mg/dL)				
N	53	55	58	58
Mean (SD)	−0.2 (7.3)	3.3 (7.5)**^†^	3.9 (11.0)**^†^	4.5 (8.6)***^††^
Triglycerides (mg/dL)				
N	53	55	58	58
Mean (SD)	−21.5 (206.2)	−20.7 (119.1)	−23.9 (83.8)*	−44.5 (95.1)***
Adiponectin (μg/mL)				
N	53	55	58	58
Mean (SD)	−0.17 (0.99)	0.71 (1.11)***^†††^	0.96 (1.61)***^†††^	0.45 (2.73)
Systolic blood pressure (mmHg)				
N	56	57	58	58
Mean (SD)	−3.2 (13.1)	−6.8 (13.1)***	−7.6 (11.4)***	−9.4 (11.1)***^††^
Diastolic blood pressure (mmHg)				
N	56	57	58	58
Mean (SD)	−1.4 (9.8)	−5.6 (9.8)***^†^	−4.1 (8.4)***	−4.1 (8.3)***
Waist circumference (cm)				
N	53	55	58	58
Mean (SD)	0.02 (3.95)	−2.42 (3.37)***^†††^	−2.47 (2.99)***^†††^	−2.27 (3.22)***^††^

*P < 0.05, **P < 0.01, and ***P < 0.001 vs. baseline.

^†^*P <* 0.05, ^††^*P <* 0.01, and ^†††^*P <* 0.001 vs. placebo.

Abbreviations: *LS mean* least squares mean, *95% CI* 95% confidence interval, *NGSP* National Glycohemoglobin Standardization Program, *HOMA-β* Homeostatic model assessment of β cell function, *HOMA-IR* Homeostatic model assessment of insulin resistance, *2-h PPG* 2-hour postprandial glucose*.*

### Secondary endpoints

**Figure 3:**
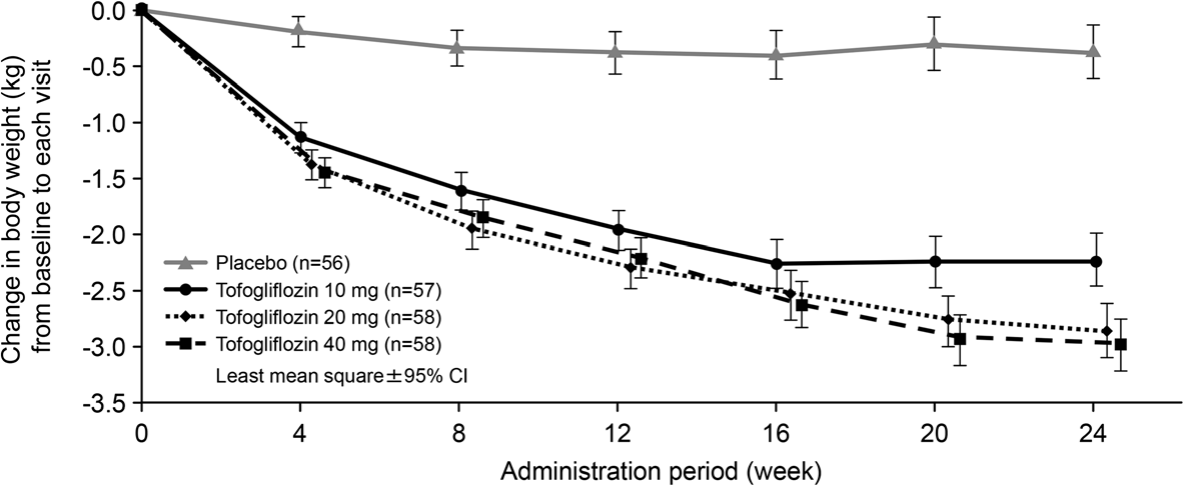
**Mean change (LS mean) in body weight from baseline to time of visit.** Mean changes (LS mean and 95% CI) in body weight from baseline to time of visit for each treatment group (placebo, tofogliflozin 10, 20 or 40 mg) in the FAS (with LOCF) were plotted. Mean change from baseline in body weight at week 24 (LS mean) and LS mean difference versus placebo are shown in the table below the graph. LS means, LS mean differences and 95% CI were estimated using an ANCOVA model with treatment groups (placebo and tofogliflozin 10, 20, or 40 mg) as a fixed effect and baseline body weight as a covariate. Among the tofogliflozin groups, the tofogliflozin 40 mg group had the greatest decrease.

**Figure 4:**
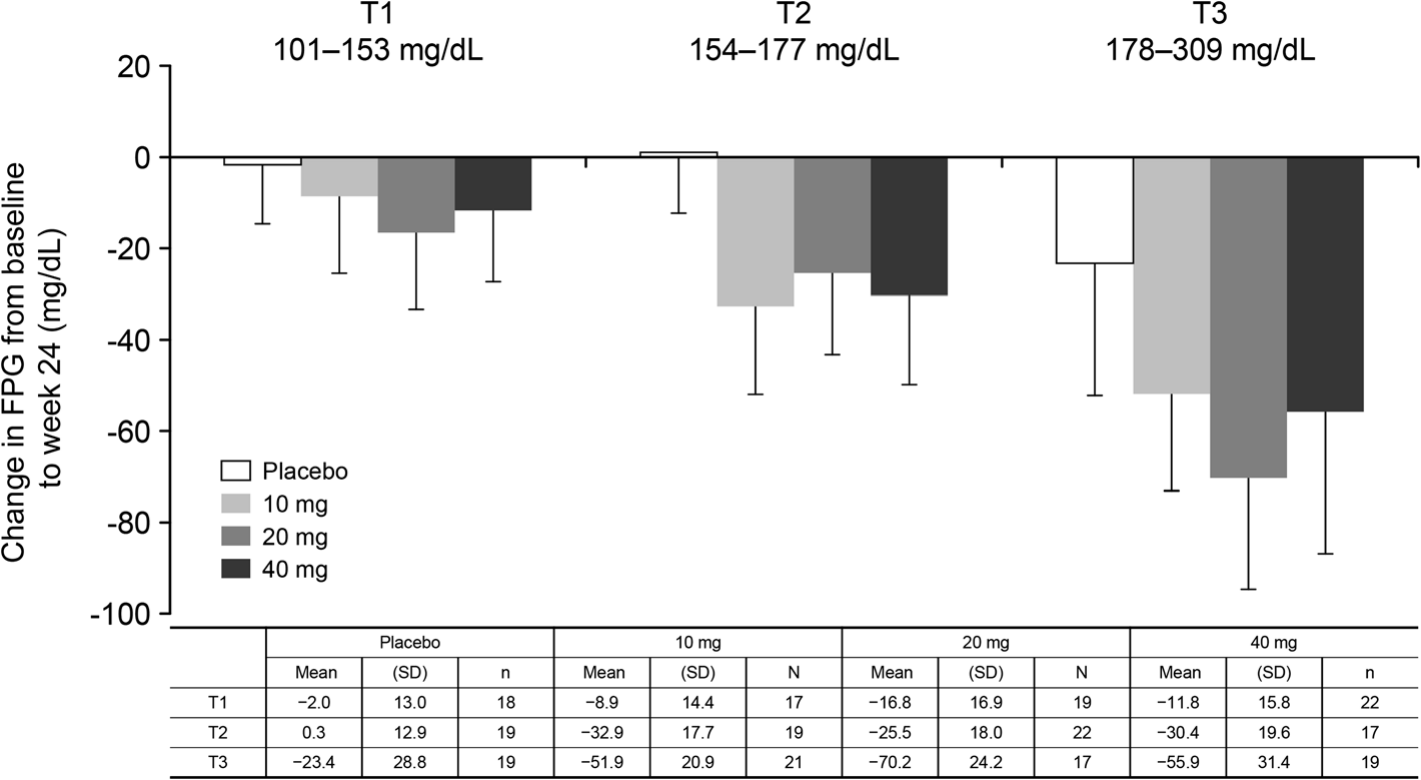
**Mean changes in fasting plasma glucose from baseline to week 24 by baseline fasting blood glucose tertile.** The mean change in fasting blood glucose from baseline to week 24 tended to increase with higher baseline fasting blood glucose tertile (T).

The secondary endpoints are summarized in Table 3. The decreases in fasting blood glucose from baseline to week 24 were significantly greater in all tofogliflozin groups than in the placebo group, with placebo-adjusted differences of −23.307, −27.338, and −23.766 mg/dL for tofogliflozin 10 mg, 20 mg, and 40 mg, respectively. The reductions in body weight were also significantly greater in the tofogliflozin groups, with placebo-adjusted differences of −1.87, −2.50, and −2.61 kg for tofogliflozin 10 mg, 20 mg, and 40 mg, respectively. Among the tofogliflozin groups, the tofogliflozin 20 mg group showed the greatest decrease in fasting blood glucose, and the tofogliflozin 40 mg group showed the greatest decrease in body weight (Table 3 and Figure 3). When we divided patients into tertiles of baseline values, the change in fasting blood glucose from baseline to week 24 was greater in patients with higher baseline fasting blood glucose (Figure 4).

### Other secondary endpoints

Fasting and 2-h postprandial insulin levels decreased significantly in the tofogliflozin groups compared with the placebo group; however, the magnitudes of the reductions were small (Table 3). Systolic and diastolic blood pressures decreased significantly in the tofogliflozin groups relative to the placebo group (Table 3). There were significant improvements in high-density lipoprotein cholesterol and triglyceride levels in all of the tofogliflozin groups. Although total cholesterol and LDL-C levels decreased slightly in the tofogliflozin 10 and 20 mg groups, the magnitudes of the reductions were less than those in the placebo group. However, there was a non-significant increase in total cholesterol level and a significant increase in LDL-C level in the tofogliflozin 40 mg group. The decrease in 2-h postprandial glucose and waist circumference from baseline to week 24 was significantly greater in all tofogliflozin groups than in the placebo group.

### Adverse events

Table 4 presents a summary of the AEs and ADRs, along with AEs occurring at an incidence of ≥5% in any treatment group. Hyperketonemia, ketonuria, pollakiuria, and headache were more likely to occur in the tofogliflozin groups than in the placebo group. Dose-dependent AEs were hyperketonemia and pollakiuria. All events were mild, required no treatment, and resolved or improved, except for one event in one patient, for which follow-up was unnecessary.

**Table 4:** Summary of adverse events and adverse drug reactions (Safety population)

	**Placebo**	**Tofogliflozin**		
		**10 mg**	**20 mg**	**40 mg**
n	56	58	58	58
Number of patients with AEs	25 (44.6%)	35 (60.3%)	31 (53.4%)	31 (53.4%)
Number of AEs	40	80	62	69
Number of patients with ADRs	4 (7.1%)	16 (27.6%)	15 (25.9%)	16 (27.6%)
Number of ADRs	6	24	26	24
Deaths	0 (0.0%)	0 (0.0%)	0 (0.0%)	0 (0.0%)
Serious AEs	2 (3.6%)*	2 (3.4%)†	0 (0.0%)	3 (5.2%)‡
Serious ADRs	0 (0.0%)	0 (0.0%)	0 (0.0%)	1 (1.7%)§
AEs leading to discontinuation	1 (1.8%)	1 (1.7%)	0 (0.0%)	2 (3.4%)
ADRs leading to discontinuation	0 (0.0%)	1 (1.7%)	0 (0.0%)	1 (1.7%)
AEs occurring in more than ≥5% of patients in any treatment groups				
Infections and infestations				
Nasopharyngitis	12 (21.4%)	9 (15.5%)	6 (10.3%)	6 (10.3%)
Upper respiratory tract infection	1 (1.8%)	0 (0.0%)	3 (5.2%)	2 (3.4%)
Investigations				
Hyperketonemia	1 (1.8%)	2 (3.4%)	7 (12.1%)	8 (13.8%)
Ketonuria	0 (0.0%)	1 (1.7%)	3 (5.2%)	0 (0.0%)
Increased urinary β-2 microglobulin	1 (1.8%)	3 (5.2%)	0 (0.0%)	0 (0.0%)
Renal and urinary disorders				
Pollakiuria	1 (1.8%)	3 (5.2%)	4 (6.9%)	6 (10.3%)
Musculoskeletal and connective tissue disorders				
Back pain	3 (5.4%)	3 (5.2%)	2 (3.4%)	1 (1.7%)
Nervous system disorders				
Headache	0 (0.0%)	3 (5.2%)	0 (0.0%)	1 (1.7%)
Other events of special interest in <5% of patients				
Genitourinary infections				
Cystitis	0 (0.0%)	0 (0.0%)	0 (0.0%)	1 (1.7)
Vulvitis	0 (0.0%)	0 (0.0%)	0 (0.0%)	1 (1.7)
Hypoglycemia	0 (0.0%)	1 (1.7)	0 (0.0%)	1 (1.7)
Increased urine volume	0 (0.0%)	0 (0.0%)	1 (1.7)	1 (1.7)
Volume-related events				
Orthostatic hypotension	0 (0.0%)	0 (0.0%)	0 (0.0%)	1 (1.7)
Dizziness	1 (1.8%)	2 (3.4%)	0 (0.0%)	0 (0.0%)
Postural dizziness	0 (0.0%)	1 (1.7)	1 (1.7)	0 (0.0%)

*Fibular fracture in one patient; colon and esophageal cancer in one patient.

^†^Meniscus disorder in one patient; unconsciousness in one patient.

^‡^Myocardial ischemia in one patient; gastrointestinal interstitial cancer in one patient; whiplash and contusion in one patient.

^§^Myocardial ischemia in one patient.

Abbreviations: *AE* Adverse event, *ADR* Adverse drug reaction.

There were no deaths. However, myocardial ischemia occurred in one patient in the tofogliflozin 40 mg group, and this was classified as a serious ADR. The patient had dyslipidemia, hypertension and obesity, which are considered as risk factors for the progression of arteriosclerosis. However, because the event occurred during the treatment with tofogliflozin, a causal relationship to tofogliflozin could not be excluded. Because the patient showed improvements in glycemic control during the treatment with tofogliflozin, the administration of tofogliflozin was continued.

Among AEs leading to discontinuation, mild vertigo in one patient in the tofogliflozin 10 mg group and moderate weight decrease in one patient in the tofogliflozin 40 mg group were classified as ADRs. Both events resolved.

The severity of AEs related to hypoglycemia was mild or moderate, and all events resolved within a day. Urinary tract and genital infections occurred in different patients in the tofogliflozin 40 mg group (1.7%). These infections were cystitis and vulvitis. Both events were mild and resolved without treatment. The episode of vulvitis was not considered related to tofogliflozin.

### Laboratory variables

Table 5 presents the change from baseline to week 24 in laboratory parameters. There were no abnormal changes in any laboratory variable of ≥5% in any of the tofogliflozin groups compared with placebo. Mean blood urea nitrogen, total serum ketones, acetoacetic acid, β-hydroxybutyric acid, and magnesium values increased over time in the tofogliflozin groups but not in the placebo group, while uric acid, aspartate aminotransferase, alanine aminotransferase, and γ-glutamyl transpeptidase levels decreased significantly in the tofogliflozin groups compared with the placebo group (Table 5).

**Table 5:** Laboratory variables at baseline and change from baseline to week 24 (Safety population)

	**Placebo**		**Tofogliflozin**					
			**10 mg**		**20 mg**		**40 mg**	
	**Baseline**	**Change**	**Baseline**	**Change**	**Baseline**	**Change**	**Baseline**	**Change**
n	56	48	58	54	58	56	58	54
Total serum ketones (μmol/L), Mean (SD)	87.4 (70.89)	29.7 (123.95)	93.2 (85.12)	45.6 (116.53)**	115.1 (144.01)	59.5 (136.24) **	127.9 (155.47)	141.2 (253.68) ***^††^
Acetoacetic acid (μmol/L), Mean (SD)	26.8 (16.66)	7.1 (30.83)	29.0 (21.52)	10.7 (30.35)*	34.5 (37.49)	14.8 (32.49) **	40.1 (40.69)	31.0 (63.09) ***^††^
β-HBA (μmol/L), Mean (SD)	60.7 (55.07)	22.6 (93.93)	64.3 (65.39)	34.7 (87.14)**	80.6 (107.30)	44.7 (105.67) **	88.0 (116.29)	110.0 (194.65)***^††^
AST (IU/L, 37°C), Mean (SD)	26.5 (10.48)	0.1 (8.85)	25.6 (11.15)	−3.2 (8.22)**	26.1 (10.04)	−3.2 (8.23)**	26.6 (10.63)	−3.7 (8.88)**^†^
ALT (IU/L, 37°C), Mean (SD)	28.6 (15.62)	−0.4 (10.95)	28.7 (19.87)	−6.5 (11.56)***^†^	30.10 (18.59)	−7.7 (12.37)***^††^	29.8 (17.51)	−6.8 (12.91)***^††^
γ-GTP (IU/L, 37°C), Mean (SD)	51.3 (56.47)	−3.2 (31.69)	44.2 (32.25)	−9.7 (22.44) **	52.2 (60.37)	−17.0 (33.31) ***^†^	55.3 (89.75)	−17.3 (56.53)*
BUN (mg/dL), Mean (SD)	14.8 (3.4)	−0.2 (2.7)	14.9 (3.3)	1.5 (3.6) **^††^	14.6 (4.2)	1.5 (4.1) **^†^	14.2 (3.2)	1.8 (3.5) ***^††^
SCr (mg/dL), Mean (SD)	0.72 (0.18)	−0.02 (0.06)	0.70 (0.15)	−0.00 (0.06)	0.71 (0.21)	−0.02 (0.15)	0.70 (0.16)	0.02 (0.06)*^††^
eGFR (mL/min/1.73 m^2^), Mean (SD)	83.78 (17.68)	2.53 (9.10)	84.68 (20.04)	0.41 (8.48)	86.78 (19.62)	1.23 (12.89)	86.00 (18.18)	−1.97 (7.97)^††^
Uric acid (mg/dL), Mean (SD)	5.09 (1.39)	0.10 (0.67)	4.77 (1.01)	−0.30 (0.68) **^††^	5.01 (1.19)	−0.33 (0.78) **^††^	5.14 (1.27)	−0.14 (1.08)

*P < 0.05, **P < 0.01, and ***P < 0.001 vs. baseline.

^†^*P <* 0.05, ^††^*P <* 0.01, and ^†††^*P <* 0.001 vs. placebo.

Abbreviations: *β-HBA* β-hydroxybutyrate, *AST* aspartate aminotransferase, *ALT* alanine aminotransferase, *GTP* glutamyl transpeptidase, *BUN* blood urea nitrogen, *SCr* serum creatinine, *eGFR estimated * glomerular filtration rate.

## Discussion

In this study, we investigated the effects of tofogliflozin, a selective SGLT2 inhibitor, on HbA_1c_ in adults with type 2 diabetes mellitus inadequately controlled with diet and exercise. We observed a statistically significant decrease in HbA_1c_, the primary endpoint, with all three doses of tofogliflozin as compared with placebo after 24 weeks of treatment. Among the tofogliflozin groups, the 20 mg dose had the greatest effect, reducing HbA_1c_ by 1.017%. These improvements in HbA_1c_ occurred in conjunction with significant reductions in fasting blood glucose in all three tofogliflozin groups. Fasting blood glucose was also decreased in the two recent canagliflozin studies, but showed a slight progressive decrease after the maximal reduction was observed at weeks 4 [5] and 6 [6]. In the present study, although the median plasma trough concentration of tofogliflozin increased in a dose-dependent manner (data not shown), this was not reflected by dose-dependent changes in HbA_1c_ or fasting blood glucose. In a prior pharmacodynamic study, the cumulative daily urine glucose excretion was comparable at doses of 10, 20, or 40 mg tofogliflozin (unpublished data). Therefore, it is likely SGLT2 inhibition is saturated at tofogliflozin doses of 10 mg or above, preventing further improvements in HbA_1c_ and other glycemic variables at doses exceeding 10 mg.

The decrease in body weight from baseline to week 24 ranged from 2.23 to 2.97 kg with tofogliflozin, which is comparable to the level of weight loss seen with dapagliflozin after 24 weeks [7], and similar to that achieved with glucagon-like peptide-1 analogs [8]. We also observed a decrease in waist circumference of approximately 2 cm in the tofogliflozin groups, suggesting that tofogliflozin may improve metabolic disorders. Again, this decrease is similar to that observed with dapagliflozin [7].

Tofogliflozin also tended to reduce systolic and diastolic blood pressure to a greater extent than placebo, and improved high-density lipoprotein–cholesterol and triglyceride levels. A slight decrease in uric acid was also observed. The reductions in total cholesterol and LDL-C levels were slightly smaller in the tofogliflozin groups than in the placebo group, and there was a non-significant increase in total cholesterol level and a significant increase in LDL-C level in the 40 mg group compared with the placebo group. Other recent studies of canagliflozin and dapagliflozin have also described modest, dose-related increases in LDL-C levels [5,6,9], suggesting SGLT2 inhibitors have limited effects on total cholesterol or LDL-C. Taken together, these findings indicate that tofogliflozin may ameliorate some of the metabolic abnormalities associated with type 2 diabetes, although prospective, long-term studies are needed to confirm this possibility.

The results of two Phase 3 studies of tofogliflozin were jointly reported in article by Tanizawa et al. [10]. In these studies, Japanese patients were treated with 20 or 40 mg tofogliflozin as monotherapy or in combination with other oral antidiabetic agents for 52 weeks. These doses were selected based on the clinical efficacy and tolerability of the three doses used in the present study. As in the present study, both doses in the Phase 3 studies were well tolerated, with a treatment discontinuation rate of <6% over 52 weeks. The mean reductions in HbA1c were 0.67% and 0.66% in the 20 and 40 mg groups, respectively, as monotherapy. In the combination study, the mean HbA1c reduction ranged from 0.71% to 0.93% across the subgroups by dose and background therapy.

A recent meta-analysis of 45 placebo-controlled studies and 13 active-controlled studies [11] revealed that SGLT2 inhibitors significantly reduced HbA_1c_ relative to placebo (mean difference vs. placebo: 0.66%; 95% CI −0.73 to −0.58%), although the change was similar to that observed with other drugs (mean difference vs. other drugs: −0.06%; 95% CI: −0.18 to 0.05%). The analysis also revealed that SGLT2 inhibitors reduced body weight (mean difference vs. other drugs: −1.80 kg; 95% CI −3.50 to −0.11 kg) and systolic blood pressure (mean difference vs. other drugs: −4.45 mmHg; 95% CI: −5.73 to −3.18 mmHg). Therefore, the results of this study are quite consistent with those reported for other SGLT2 inhibitors in terms of the improvements in glycemic control, body weight, and systolic blood pressure.

The main AEs were hyperketonemia, ketonuria, and pollakiuria. The latter occurred at an incidence of ≥5% with tofogliflozin, suggesting that appropriate fluid supplementation is important. Serious AEs and AEs that led to discontinuation occurred at similar rates in the tofogliflozin and placebo groups, and did not appear to be dose-dependent. Hypoglycemia was observed in the tofogliflozin groups, but not in the placebo group, although the relationship was not dose-dependent. However, the incidence of hyperketonemia and pollakiuria in the tofogliflozin groups appeared to be dose-dependent. Cystitis and vulvitis were observed in one patient each in the tofogliflozin groups. However, both events were classified as mild. Thus, the AE profile of tofogliflozin was similar to that of SGLT2 inhibitors in general, as reported in the earlier meta-analysis [11]. Significant decreases were observed in aspartate aminotransferase, alanine aminotransferase, and γ-glutamyl transpeptidase, suggestive of improvements in liver function during the study, although this warrants confirmation in future studies.

Several SGLT2 inhibitors have been developed, including canagliflozin, dapagliflozin, ipragliflozin, and empagliflozin, and some have been approved in various regions for the treatment of type 2 diabetes (e.g., canagliflozin in the US, dapagliflozin in Europe, and ipragliflozin in Japan). Unlike other agents, they reduce hyperglycemia by enhancing urinary excretion of excess glucose. Because hypoglycemia, weight gain, and worsening of glycemic control during long-term usage are of concern in relation to many currently available antihyperglycemic agents [1,2], the development of SGLT2 inhibitors, which are not associated with hypoglycemia or weight gain, is important.

SGLT2 is exclusively expressed in the kidneys [12,13] and families with hereditary dysfunction in SGLT2 due to genetic defects develop familial renal glucosuria. Although up to 100 g of glucose is excreted in urine daily, this does not prevent the majority of patients with familial glucosuria from living a normal life [14,15]. Conversely, mutations in the SGLT1 gene, involved in glucose absorption in kidney and gastrointestinal tract, may cause glucose–galactose malabsorption, characterized by serious diarrhea [16]. Thus, inhibition of SGLT1 may cause gastrointestinal disorders, and therefore it is desirable to develop highly selective drugs for SGLT2 that specifically inhibit renal glucose reabsorption without affecting intestinal glucose handling.

Tofogliflozin is a SGLT2 inhibitor, with 2,900-fold greater selectivity for SGLT2 than SGLT1, and has the highest selectivity of all clinically developed inhibitors [17]. This potent selectivity might have contributed to relatively few AEs in this clinical trial. Furthermore, the reduced risk of hypoglycemia with tofogliflozin might result from its high selectivity for SGLT2, as it does not inhibit glucose reabsorption by SGLT1. More specifically, in a hyperglycemic state, tofogliflozin promotes the urinary excretion of excessive glucose from the glomerular filtrate by inhibiting SGLT2, leading to a decrease in blood glucose. Importantly, because of this high selectivity for SGLT2, glucose is reabsorbed by SGLT1 at a low plasma glucose level, and thus tofogliflozin has low risk of hypoglycemia, unlike other currently available type 2 diabetes medications. Although tofogliflozin promoted a decrease in fasting blood glucose, safety issues must be continuously and carefully monitored after product launch. Like other SGLT2 inhibitors [18], tofogliflozin was associated with significant reductions in body weight compared with placebo. These body weight reductions might be associated with a compensatory reduction of visceral fat resulting from the loss of calories following increased urinary glucose excretion [15]. Indeed, a dapagliflozin study suggested that the main source of this body weight reduction is visceral fat tissue [7]. An increase in blood ketone bodies was frequently detected, although no clinical symptoms related to this increase were reported. This increase in ketone levels might be due to an increase in lipolysis and mobilization of lipids/free fatty acids to compensate for the loss of glucose in urine. The increase in lipid metabolism might contribute to reductions in visceral fat and body weight. The influence of increased blood ketone bodies on the safety of the study drug is unknown. Because the decreases in fasting insulin levels in the tofogliflozin groups were small, the hyperketonemia was unlikely to be due to diabetic ketoacidosis caused by extreme insulin deficiency.

There were some limitations in this study that warrant mention. First, the number of type 2 diabetes mellitus patients enrolled was relatively low compared with two recent canagliflozin studies (230 vs 383 and 584) [5,6]. Second, a longer study duration might have been more beneficial; however, the study period of 24 weeks was longer than that of Inagaki et al. (12 weeks) [5] and similar to that of Stenlof et al. (26 weeks) [6]. In addition, the results of two Phase 3 studies were recently reported, and showed good efficacy and tolerability profiles of 20 and 40 mg tofogliflozin as monotherapy or combination therapy over 52 weeks of treatment [10]. Of course, it will also be interesting to see the longer-term efficacy and safety of tofogliflozin alone or in combination with other oral antidiabetic drugs, such as in the ongoing 4-year EMPA-REG H2H-SU study, in which patients are being treated with empagliflozin or glimepiride as an add-on to metformin [19]. Third, the study was limited to Japanese patients treated in outpatient settings, limiting its generalizability to patients in other countries. Fourth, we did not consider the effects of renal impairment; future studies should examine the impact of renal impairment on the efficacy and safety of tofogliflozin. Although further efficacy and safety data are needed, because tofogliflozin acts independently from insulin, there is little risk of increasing the burden on the pancreas and causing hypoglycemia. Its ability to enhance glycemic control and reduce body weight is also clinically important. Moreover, the ability of tofogliflozin to reduce blood pressure, improve lipid metabolism, and reduce uric acid levels suggests that it may also help to prevent vascular complications associated with type 2 diabetes mellitus. SGLT2 inhibitors may also improve hepatic triglyceride accumulation, as observed with dapagliflozin [7]. Further studies and real-life observation are needed to confirm the efficacy and safety of tofogliflozin and other SGLT2 inhibitors in clinical practice.

## Conclusion

Tofogliflozin 10, 20, or 40 mg administered orally once daily for 24 weeks significantly decreased HbA_1c_, fasting blood glucose, and body weight as compared with placebo in patients with type 2 diabetes mellitus. Tofogliflozin was also generally well tolerated, with predominantly mild AEs being experienced. Considering the efficacy and safety profile of tofogliflozin, doses of 20 or 40 mg, as used in the recent Phase 3 studies [10], might be appropriate for clinical use in Japanese patients. Further studies might be needed to confirm these findings, especially compared with other oral agents. Our results and the results of the recent Phase 3 studies provide support for using tofogliflozin as either monotherapy or in combination with other oral antidiabetic drugs.

## Abbreviations

ADR: Adverse drug reaction; AE: Adverse event; ANCOVA: Analysis of covariance; BMI: Body mass index; FAS: Full analysis set; HbA1c: Hemoglobin A1c; HOMA-β: Homeostatic model assessment of β cell function; HOMA-IR: Homeostatic model assessment of insulin resistance; LDL-C: Low-density lipoprotein–cholesterol; NGSP: National Glycohemoglobin Standardization Program; PPS: Per-protocol set; SD: Standard deviation; SGLT: Sodium/glucose cotransporter.

## Competing interests

Kohei Kaku has received honoraria for lectures from MSD K.K., Novartis Pharma K.K., Takeda Pharmaceutical Company Limited, Mitsubishi Tanabe Pharma Corporation, Daiichi Sankyo Co., Ltd., Kowa Pharmaceutical Co. Ltd., Novo Nordisk Pharma Ltd., Sanofi K.K., and Dainippon Sumitomo Pharma Co., and unrestricted grants from MSD K.K., Nippon Boehringer Ingelheim Co., Ltd., Novartis Pharma K.K., Takeda Pharmaceutical Company Limited, Mitsubishi Tanabe Pharma Corporation, Daiichi Sankyo Co., Ltd., Novo Nordisk Pharma Ltd., Sanofi K.K., Dainippon Sumitomo Pharma Co., Astellas Pharma Inc., and AstraZeneca K.K.

Hirotaka Watada has received lecture fees from Nippon Boehringer Ingelheim Co., Ltd., Sanofi K.K., Ono Pharmaceutical Co., Ltd., Novo Nordisk Pharma Ltd., Novartis Pharma K.K., Eli Lilly Japan K.K., Sanwa Kagaku Kenkyusho Co., Ltd., Daiichi Sankyo Co., Ltd., Takeda Pharmaceutical Company Limited, MSD K.K., Dainippon Sumitomo Pharma Co., and Kowa Company, Ltd., and research funding from Nippon Boehringer Ingelheim Co., Ltd., Pfizer Japan Inc., Mochida Pharmaceutical Co., Ltd., Sanofi K.K., Novo Nordisk Pharma Ltd., Novartis Pharma K.K., Sanwa Kagaku Kenkyusho Co., Ltd., Terumo Corporation, Eli Lilly Japan K.K., Mitsubishi Tanabe Pharma Corporation, Daiichi Sankyo Co., Ltd., Takeda Pharmaceutical Company Limited, MSD K.K., Shionogi & Co., Ltd., Dainippon Sumitomo Pharma Co., Kissei Pharmaceutical Co., Ltd., and AstraZeneca K.K.

Yasuhiko Iwamoto has received honoraria for lectures from MSD K.K., Sanofi K.K., and Eli Lilly Japan K.K.

Kazunori Utsunomiya has received honoraria for lectures from MSD K.K., Ono Pharmaceutical Co., Ltd., Nippon Boehringer Ingelheim Co., Novartis Pharma K.K., Takeda Pharmaceutical Company Limited, Mitsubishi Tanabe Pharma Corporation, Daiichi Sankyo Co., Ltd., Sanwa Kagaku Kenkyusho Co., Ltd., Kowa Pharmaceutical Co. Ltd., Novo Nordisk Pharma Ltd., Eli Lilly Japan K.K., Sanofi K.K., Dainippon Sumitomo Pharma Co., Ltd., Shionogi & Co., Ltd., Kissei Pharmaceutical Co., Ltd., Bayer Yakuhin, Ltd., Astellas Pharma Inc., Pfizer Japan Inc., AstraZeneca K.K., Chugai Pharmaceutical Co., Ltd., Teijin Pharma Limited., Mochida Pharmaceutical Co., Ltd., Roche Diagnostics K.K., and Johnson & Johnson K.K., and unrestricted grants from MSD K.K., Ono Pharmaceutical Co., Ltd., Nippon Boehringer Ingelheim Co., Ltd., Novartis Pharma K.K., Takeda Pharmaceutical Company Limited, Mitsubishi Tanabe Pharma Corporation, Daiichi Sankyo Co., Ltd., Sanwa Kagaku Kenkyusho Co., Ltd., Kowa Pharmaceutical Co. Ltd., Novo Nordisk Pharma Ltd., Eli Lilly Japan K.K., Sanofi K.K., Dainippon Sumitomo Pharma Co., Ltd., Shionogi & Co., Ltd., Kissei Pharmaceutical Co., Ltd., Bayer Yakuhin, Ltd., Astellas Pharma Inc., and AstraZeneca K.K.

Yasuo Terauchi has received honoraria for lectures from MSD K.K., Ono Pharmaceutical Co., Ltd., Nippon Boehringer Ingelheim Co., Ltd., Novartis Pharma K.K., Takeda Pharmaceutical Company Limited, Mitsubishi Tanabe Pharma Corporation, Daiichi Sankyo Co., Ltd., Sanwa Kagaku Kenkyusho Co., Ltd., Kowa Pharmaceutical Co. Ltd., Novo Nordisk Pharma Ltd., Eli Lilly Japan K.K., Sanofi K.K., Dainippon Sumitomo Pharma Co., Ltd., Shionogi & Co., Ltd., Kissei Pharmaceutical Co., Ltd., Bayer Yakuhin, Ltd., Astellas Pharma Inc., Pfizer Japan Inc., AstraZeneca K.K., Chugai Pharmaceutical Co., Ltd., Teijin Pharma Limited., Mochida Pharmaceutical Co., Ltd., Roche Diagnostics K.K., and Johnson & Johnson K.K., and unrestricted grants from MSD K.K., Ono Pharmaceutical Co., Ltd., Nippon Boehringer Ingelheim Co., Ltd., Novartis Pharma K.K., Takeda Pharmaceutical Company Limited, Mitsubishi Tanabe Pharma Corporation, Daiichi Sankyo Co., Ltd., Sanwa Kagaku Kenkyusho Co., Ltd., Kowa Pharmaceutical Co. Ltd., Novo Nordisk Pharma Ltd., Eli Lilly Japan K.K., Sanofi K.K., Dainippon Sumitomo Pharma Co., Ltd., Shionogi & Co., Ltd., Kissei Pharmaceutical Co., Ltd., Bayer Yakuhin, Ltd., Astellas Pharma Inc., and AstraZeneca K.K.

Kazuyuki Tobe has received honoraria for lectures from Takeda Pharmaceutical Company Limited and Mitsubishi Tanabe Pharma Corporation, and scholarship funds from Novo Nordisk Pharma Ltd., Novartis Pharma K.K., Ono Pharmaceutical Co., Ltd., MSD K.K., Astellas Pharma Inc., Sanofi K.K., Iryouhoujin Shichitokukai, Seida Sadayoshi, Mitsubishi Tanabe Pharma Corporation, Takeda Pharmaceutical Company Limited, and Chugai Pharmaceutical Co., Ltd.

Yukio Tanizawa has received honoraria for lectures from Novartis Pharma K.K., Takeda Pharmaceutical Company Limited, MSD K.K., Ono Pharmaceutical Co., Ltd., Sanofi K.K., Mitsubishi Tanabe Pharma Corporation, Novo Nordisk Pharma Ltd., Nippon Boehringer Ingelheim Co., Ltd., and Dainippon Sumitomo Pharma Co., Ltd., and scholarship funds from Kowa Pharmaceutical Co. Ltd., Dainippon Sumitomo Pharma Co., Takeda Pharmaceutical Company Limited, MSD K.K., Astellas Pharma Inc., Daiichi Sankyo Co., Ltd., Kyowa Hakko Kirin Co., Ltd., Sanofi K.K., Novartis Pharma K.K., and Nippon Boehringer Ingelheim Co., Ltd.

Eiichi Araki has received honoraria for lectures from MSD K.K., Sanofi K.K., Daiichi Sankyo Co., Ltd., Mitsubishi Tanabe Pharma Corporation, Eli Lilly Japan K.K., Novo Nordisk Pharma Ltd., Nippon Boehringer Ingelheim Co., Ltd., Novartis Pharma K.K., Kowa Pharmaceutical Co. Ltd., and Kowa Company, Ltd., and scholarship funds from Mitsubishi Tanabe Pharma Corporation, Sanofi K.K., Nippon Boehringer Ingelheim Co., Ltd., Astellas Pharma Inc., Dainippon Sumitomo Pharma Co., Ltd., Pfizer Japan Inc., and MSD K.K.

Masamichi Ueda is an employee of Chugai Pharmaceutical Co., Ltd.

Hideki Suganami is an employee of Kowa Company, Ltd.

Daisuke Watanabe is an employee of Sanofi K.K.

## Authors’ contributions

KK contributed to the conception, organization and execution of the study; interpretation of data; writing of the first draft; and revised the manuscript for important intellectual content. HW contributed to the conception, organization and execution of the study; the collection and interpretation of data; and revised the manuscript for important intellectual content. YI contributed to the conception, organization and execution of the study; the collection and interpretation of data; and revised the manuscript for important intellectual content. KU contributed to the conception, organization and execution of the study; the collection and interpretation of data; and revised the manuscript for important intellectual content. Y Terauchi contributed to the conception, organization and execution of the study; the interpretation of data; and revised the manuscript for important intellectual content. KT contributed to the conception, organization and execution of the study; the collection and interpretation of data; and revised the manuscript for important intellectual content. Y Tanizawa contributed to the conception, organization and execution of the study; the collection and interpretation of data; and revised the manuscript for important intellectual content. EA contributed to the conception, organization and execution of the study; the interpretation of data; and revised the manuscript for important intellectual content. MU created the database, performed statistical analyses, and contributed to the interpretation of the data. HS created the database, performed statistical analyses, and contributed to the interpretation of the data. DW created the database, performed statistical analyses, and contributed to the interpretation of the data. All authors have read and approved the final manuscript for submission.

## Supplementary Material

Additional file 1: Table S1 Study schedule and tests. Click here for file

## Members of the coordinating committee

Kazunori Utsunomiya (Division of Diabetes, Metabolism and Endocrinology, Department of Internal Medicine, Jikei University School of Medicine, Tokyo), Yasuhiko Iwamoto (Tokyo Women’s Medical University School of Medicine, Tokyo), Kazuyuki Tobe (First Department of Internal Medicine, University of Toyama, Toyama), Yukio Tanizawa (Internal Medicine III, Yamaguchi University Hospital, Yamaguchi), Hirotaka Watada (Department of Endocrinology & Metabolism, Juntendo University Graduate School of Medicine, Tokyo), Yasuo Terauchi (Department of Endocrinology and Metabolism, Graduate School of Medicine, Yokohama City University Hospital, Yokohama), and Eiichi Araki (Department of Metabolic Medicine, Faculty of Life Sciences, Kumamoto University, Kumamoto).

## List of participating institutions

General Incorporated Foundation Ohta General Hospital Ohta Nishinouchi Hospital, Center for Diabetes; HDC Atlas Clinic, Endocrinology and Metabolism; Japanese Red Cross Nagoya Daini Hospital, Department of Diabetes and Endocrinology; Jikei University Hospital, Department of Diabetes, Metabolism and Endocrinology; Medical Corporation Asanakai Tokyo-Eki Center-building Clinic, Internal Medicine; Medical Corporation Doujin Memorial Meiwa Hospital, Internal Medicine; Medical Corporation Jinnouchikai Jinnouchi Hospital, Internal Medicine; Medical Corporation Junshinkai Ageo Medical Clinic; Medical Corporation Kunwakai Aiwa Clinic of Internal Medicine; Medical Corporation Lifestyle Tomonaga Clinic, Internal Medicine; Medical Corporation Manda Memorial Hospital, Internal Medicine; Medical Corporation Mitsuyoshikai Tokyo-Ekimae Building Clinic, Internal Medicine; Medical Corporation Onoyuri Clinic of Internal Medicine, Internal Medicine; Medical Corporation Oyama East Clinic, Internal Medicine; Medical Corporation Rakuseikai Okuguchi Clinic of Internal Medicine; Medical Corporation Seikeikai New Tokyo Hospital, Endocrinology and Diabetes; Medical Corporation Shinanokai Shinanozaka Clinic, Internal Medicine; Medical Corporation Shinkoukai Takeuchi Hospital, Internal Medicine; Medical Corporation Shiraiwa Medical Clinic, Internal Medicine; Medical Corporation Shojukai Tenpozan Clinic of Internal Medicine, Internal Medicine; Medical Corporation Touyukai Yutenji Medical Clinic, Internal Medicine; National University Corporation Chiba University Hospital, Diabetes, Metabolism and Endocrinology; National University Corporation Kyushu University Hospital, Department of Endocrine and Metabolic Diseases/Diabetes Mellitus; National University Corporation Shimane University Hospital, Internal Medicine of Endocrinology and Metabolism; National University Corporation the University of Toyama University Hospital, First Department of Internal Medicine; Osaka City General Hospital, Metabolism and Endocrinology; Osaka City University Hospital, Center for Lifestyle Diseases and Diabetes; Social Welfare Organization Saiseikai Imperial Gift Foundation Inc., Matsuyama Hospital, Internal Medicine; Social Welfare Organization Saiseikai Imperial Gift Foundation Inc., Saiseikai Fukuoka General Hospital, Internal Medicine; Tokushima University Hospital, Internal Medicine; Tokyo Women’s Medical University Hospital, Metabolism and Diabetology; Toshiba General Hospital, Metabolism and Endocrinology; and Umeda Oak Clinic, Internal Medicine.
