# Supplementary material for: Efficacy and safety of monotherapy with the novel sodium/glucose cotransporter-2 inhibitor tofogliflozin in Japanese patients with type 2 diabetes mellitus: a combined Phase 2 and 3 randomized, placebo-controlled, double-blind, parallel-group comparative study
Source: Cardiovasc Diabetol. 2014 Mar 28;13:65. doi: 10.1186/1475-2840-13-65 (PMC4021346; doi:10.1186/1475-2840-13-65)
Supplement: Additional file 1: Table S1 — Study schedule and tests. [file 1475-2840-13-65-S1.doc]

**Supplementary Materials**

**Additional file 1: Table S**1 Study schedule and tests

| Scheduled visit | | Screening period | | Administration period (w) | | | | | | | Discontinuation | Follow-up period (w) |
| --- | --- | --- | --- | --- | --- | --- | --- | --- | --- | --- | --- | --- |
|  |  | Interim enrollment examination (max. −8 w) | Main enrollment examination ^**^ | 0 | 4 | 8 | 12 | 16 | 20 | 24 |  | 26 |
| Acceptable range for visit | |  | ± 1 w ^**^ | BL | ± 1 | ± 1 | ± 1 | ± 1 | ± 1 | ± 1 |  | ± 1 |
| Informed consent acquisition | | ○ |  |  |  |  |  |  |  |  |  |  |
| Enrollment | | ○ | ○ |  |  |  |  |  |  |  |  |  |
| Study drug administration | |  |  |  |  |  |  |  |  |  |  |  |
| Background of subject | | ○ | ○ |  |  |  |  |  |  |  |  |  |
| Body measurement | Height | ○ |  |  |  |  |  |  |  |  |  |  |
|  | Weight | ○ | ○ | ○ | ○ | ○ | ○ | ○ | ○ | ○ | ○ | ○ |
|  | Waist to hip ratio | ○ |  | ○ |  |  | ○ |  |  | ○ | ○ |  |
| Physical examination | Compliance check |  |  | ○ | ○ | ○ | ○ | ○ | ○ | ○ | ○ |  |
|  | Physical examination | ○ | ○ | ○ | ○ | ○ | ○ | ○ | ○ | ○ | ○ | ○ |
| Vital signs^*^ | | ○ |  | ○ | ○ | ○ | ○ | ○ | ○ | ○ | ○ | ○ |
| 12-lead ECG | | ○ |  | ○ |  |  | ○ |  |  | ○ | ○ |  |
| Laboratory test^†^ | Hematology | ○ |  | ○ | ○ | ○ | ○ | ○ | ○ | ○ | ○ | ○ |
|  | Serum biochemistry 1 | ○ | ○ | ○^††^ | ○ | ○ | ○ | ○ | ○ | ○^††^ | ○ | ○ |
|  | Serum biochemistry 2 | ○ |  | ○ | ○ | ○ | ○ | ○ | ○ | ○ | ○ | ○ |
|  | Serum biochemistry test 3 | ○ |  | ○ |  |  | ○ |  |  | ○ | ○ | ○ |
|  | Function test 1 | ○ |  | ○ |  |  | ○ |  |  | ○ | ○ | ○ |
|  | Function test 2 |  |  | ○ |  |  | ○ |  |  | ○ | ○ | ○ |
|  | Blood glucose-related hormones |  |  | ○^††^ |  |  | ○ |  |  | ○^††^ | ○ | ○ |
|  | Hemostasis | ○ |  | ○ | ○ |  | ○ |  |  | ○ | ○ | ○ |
|  | Urinalysis 1 | ○ |  | ○ | ○ | ○ | ○ | ○ | ○ | ○ | ○ | ○ |
|  | Urinalysis 2 | ○ |  | ○ |  |  | ○ |  |  | ○ | ○ | ○ |
| Meal loading test ^‡^ | |  |  | ○ |  |  |  |  |  | ○ |  |  |
| Visual analogue scale (sense of hunger/thirst) | | ○ |  | ○ |  |  | ○ |  |  | ○ | ○ |  |
| Self-measured blood glucose ^§^ | |  |  |  |  |  |  |  |  |  |  |  |
| Pregnancy test ^‖^ | |  | ○ |  |  |  |  |  |  | ○ | ○ |  |
| Drug concentration | |  |  | ○ |  |  | ○ |  |  | ○^¶^ | ○ |  |
| Adverse events | |  |  |  |  |  |  |  |  |  |  |  |

* Body temperature (under arm), blood pressure in the sitting position (systolic, diastolic), pulse

^†^ Hematological test: red blood cell count, hemoglobin, hematocrit, platelet count, white blood cell count, differential count (neutrophil, lymphocyte, monocyte, eosinophil, basophil)

- Serum biochemistry test 1: fasting blood glucose, HbA_1c_ (Japan Diabetes Society)
- Serum biochemistry test 2: alanine aminotransferase, aspartate aminotransferase, alkaline phosphatase, γ-glutamyl transpeptidase, total bilirubin, serum creatinine, blood urea nitrogen, lactate dehydrogenase, creatinine kinase, total protein, serum albumin, sodium, potassium, chlorine, magnesium, calcium, phosphorus, urinary acid, ketone body (total ketone body, acetoacetic acid, 3-hydroxybutyric acid)
- Serum biochemistry test 3: total cholesterol, low-density lipoprotein–cholesterol, high-density lipoprotein–cholesterol, triglycerides, free fatty acids
- Specific function test 1: high-sensitivity C-reactive protein, brain natriuretic peptide, cystatin C, bone alkaline phosphatase, intact parathyroid hormone, 25(OH) vitamin D, N-terminal telopeptide
- Specific function test 2: glycol-albumin, adiponectin
- Blood glucose-related hormones: pro-insulin, insulin, C-peptide, glucagon
- Blood coagulation test: activated partial thromboplastin time, prothrombin time (seconds and international normalized ratio), fibrinogen
- Urinalysis 1: pH, gravity, qualitative tests (protein, occult blood, ketone body, white blood cell, nitrous acid, and sediment)
- Urinalysis 2: sodium, potassium, chlorine, calcium, trace albumin, creatinine, β2-microgloblin, *N*-acetyl-β-D-glucosaminidase

^‡^ Glucose, insulin, C-peptide, glucagon, glucagon-like peptide-1 (total and active), glucagon-like peptide, 2 h-urine collection (glucose, creatinine, osmotic pressure, urinary volume)

^§^ Once a week or more, before breakfast and study drug administration

^‖^ Only for women capable of pregnancy

^¶^ Before study drug administration, before breakfast and 30, 60, 90 and 120 minutes after breakfast (five times in total)

**The main enrollment date was 4 weeks after the interim enrollment date with an acceptable range of 1 week

^††^ For fasting blood glucose and insulin, C-peptide and glucagon, a meal loading test was performed before breakfast

Abbreviations: w, weeks; BL, baseline; ECG, electrocardiogram
